# Effect of abdomen massage for prevention of feeding intolerance in preterm infants

**DOI:** 10.1186/s13052-014-0089-z

**Published:** 2014-11-14

**Authors:** Kadir Şerafettin Tekgündüz, Ayşe Gürol, Serap Ejder Apay, İbrahim Caner

**Affiliations:** Faculty of Medicine, Division of Neonatology, Atatürk University, Erzurum, Turkey; Health Services Vocational School, Atatürk University, Erzurum, Turkey; Faculty of Health Science, Atatürk University, Erzurum, 25240 Turkey

**Keywords:** Preterm infants, Abdominal massage, Feeding intolerance, Abdominal distension, Necrotizing enterocolitis

## Abstract

**Background:**

The aim of this study was to evaluate the efficacy of abdominal massage on feeding tolerance in stable preterm infants fed minimal enteral nutrition.

**Methods:**

The study was conducted on a control-grouped pre-test, post-test quasi-experimental design at the neonatal intensive care unit of a university hospital in Turkey between March and July 2012. Abdominal massage was applied to the massage group subjects for 15 minutes, 2 times daily, before the subject was fed starting in the 5-day study period.

**Results:**

The study was conducted with 27 subjects, 14 in the massage group and 13 in the control group. When frequency of defecation measurements were analysed, the difference between the first day and last day of the study was not statistically significant in the massage group. However, when daily weight gain, frequency of vomiting, abdominal circumference and gastric residual volume excess measurements were analysed, the differences between the first day and last day of the study were statistically significant in the massage group.

**Conclusions:**

In accordance with the results of the study, we suggest that nurses should apply abdominal massage twice a day as an intervention helping to prevent gastric residual volume excess and abdominal distension in enterally fed preterm infants.

## Background

Preterm infants often have feeding difficulties due to functional immaturity of the gastrointestinal (GI) tract [1]. Feeding intolerance (FI) is defined as the general incidence of high gastric residual volume (GRV), vomiting and abdominal distension. High GRV can be related to the increase in the incidence of other GI complications such as necrotizing enterocolitis (NEC) [2]. Early postnatal enteral feeding with small amounts of human milk or formula may improve the development of the GI tract, gut hormone release, and gut motility. Minimal enteral feeding has some clinical benefits, such as reducing the time to start full enteral feeding and length of hospitalisation without increasing the risk of necrotizing enterocolitis [2,3]. On the one hand, enteral FI is a major problem in premature infants, resulting in prolonged hospitalisation, increased risk of sepsis and a predisposition to serious complications due to prolonged use of parenteral nutrition [4,5]. The nutritional problems of preterm infants have become particularly relevant on short and long-term development [3]. Therefore, it is important to know and assess correctly the warning signs of the possible complications of enteral feeding [1].

The pathophysiology of FI is poorly understood, limiting the therapeutic options. Delayed gastric emptying, intestinal immaturity, ileus of prematurity and gastroesophageal reflux may all play a role. It is not clear whether one or all of these mechanisms contribute to the observed FI [6].

Research on massage has been carried out for many years. Massage has been found to increase the infant’s serotonin level, vagal activity, gastric motility; reduces stress hormone levels and colic; regulates sleeping; increases the motor development and coordination of the infant and supports weight gain [7-10]. According to a few research results, massage reduces the colic attacks of infants [7,11]. Diego et al. [8] showed the effect of a 15 minute massage per day for 5 days in preterm infants. Preterm infant massage is consistently associated with increases in vagal activity and gastric motility. In the results of another study showed that preterm infants receiving massage therapy increased vagal tone and gastric motility [12]. One hypothesis was that moderate-pressure massage stimulates vagal activity, leading to more efficient food absorption through increased gastric motility and the release of food absorption hormones [12].

The aim of this study was to evaluate the efficacy of abdominal massage on feeding tolerance in stable preterm infants fed minimal enteral nutrition in the Neonatal Intensive Care Unit (NICU). This is the first research showing that abdominal massage prevents delayed FI in enterally fed preterm infants.

## Materials and methods

### Study design

The study was conducted on a control-group pre-test, post-test quasi-experimental design at the NICU of a university hospital in Turkey between March and July 2012. Inclusion criteria included preterm infants who were being fed enterally via an orogastric tube on the NICU; gestational age 28–32 weeks; birth body weight of 1000-1750 g; without intestinal obstruction, abdominal surgery, or NEC; and had no contraindication to abdominal massage. Preterm infants were excluded from this study if they had major congenital malformations, such as congenital heart disease, gastrointestinal anomalies, hypoxic injury, respiratory failure with ventilatory support, current or previous history of NEC, suspected or confirmed sepsis.

The study was conducted with 27 subjects, 14 in the massage group and 13 in the control group. Sampling size was determined statistically by power analysis. Since there is no similar study in the literature, power analysis was calculated according to the incidence of premature birth and FI. As a result of the analysis, for a total sample size of 27, the power of study was calculated to be 99%.

### Abdominal massage application

The preterm infants received massages from the same licensed massage therapists. Abdominal massage was applied to the massage group subjects for 15 minutes, 2 times daily, before the subject was fed starting in the 5-day study period. This procedure was selected because the risk of regurgitation was low due to the emptiness of the stomach. Massage application time and frequency were decided according to studies and expert opinions found in the literature [8,12].

Moisturising lotion (Baby Lotion Natural Calm, pH 5.5; Johnson & Johnson, New Brunswick, NJ, USA) was used for massage application. Before applying massage, the infant’s abdominal circumference was measured and palpated for distension. During massage application, the subject was placed in a supine position with the head-of-bed angle elevated at 30°–45°.

The abdominal massage was applied in a clockwise direction over the intestines on the abdominal wall. The following strokes with moderate pressure were applied to the areas of the preterm infants’ abdomen:

1. Hold your hand so your pinkie finger’s edge can move like a paddle across your baby's belly. Starting at the base of the rib cage, stroke downwards with one hand and then the other in a paddle-wheel-like motion.
2. Massage the abdomen with your fingertips in a circular, clockwise motion.
3. Do the "I Love U" stroke: Trace the letter I down your baby's left side. Then trace an inverted L, stroking across the belly along the base of her ribs from her right side to her left and down. Trace an inverted U, stroking from low on the baby's right side, up and around the navel, and down the left side.
4. Walk your fingers around the navel, clockwise.
5. Hold the knees and feet together and gently press the knees up toward the abdomen. Rotate the baby's hips around a few times to the right.
6. Place your hand on the tummy horizontally and rock your hand from side to side a few times. Note: avoid massaging the tummy if the cord has not completely healed.

### Feeding protocol

In all preterm infants, orogastric tubes were inserted. A 6 Fr orogastric tube is generally inserted in these infants. Bolus feedings were given by gravity drainage after priming the tubing, every three hours in the two groups. Continuous feeds were given using an automatic syringe-pump. Gastric residuals were checked every three hours in infants in the two groups, regardless of assignment. In the unit, preterm infants were fed eight meals a day. Feeding of the preterm infants was started with a 2 or 3 ml feeding bolus for the first feeding. As food toleration develops, this amount was increased as 20 ml/kg/day and continues until it reached 140-160 ml/kg/day. During infant gavage feeding, the flow rate of milk was caused by gravity using the suspension technique without applying the pressure to the injector. Body weight was measured and recorded daily before the 9:00 a.m. feeding during the study.

The criteria accepted for food tolerance include a GRV less than half of the food amount given in the previous meal and absence of vomiting and abdominal distension.

### Data collection instruments

Research data was collected using an investigator-initiated “Follow-up Form.” Abdominal massage was provided according to the protocol “Directive on Abdominal Massage Application” based on the information found in the literature [1,2,12-15] and considering enteral feeding applications in the units where the study was conducted. The follow-up form has 12 sections including the dates and hours of follow-up, infant age, infant gender, infant’s birth weight, type of feeding, daily weight gain, GRV measurement value, abdominal circumference, the number of vomiting episodes, and frequency of defecation.

### Gastric residual volume measurement

The gastric residual volume measurement was taken before each feeding administration. A gastric residual volume measurement was made by aspirating with a 5 ml syringe before each feeding. Positioning of the gastric tube was determined by giving 0.5-1 ml of air and listening with a stethoscope at the epigastric region. The GRV measurement was then made. During the GRV measurement, the syringe piston was withdrawn slowly. When the stomach contents were no longer aspirated, the measurement was repeated to verify whether the stomach was empty.

### Collecting data from the massage group

After placing the orogastric tube in the massage group, abdominal circumference and distension were measured before initiation of the first feeding. Once feedings started, before each feeding meal, follow-up weight, vomiting, GRV measurement, abdominal circumference measurement, abdominal distension measurement (palpation), and frequency of defecation, were assessed. Abdominal massage was then administered for 15 minutes at 9:00 a.m. and 9:00 p.m. within the 5-day study period. The recorded measurements were daily documented on the “Follow-Up Form” in this period.

### Collecting data from control group subjects

Infants in the control group received feedings like the infants in the massage group. Data was collected in the same way from both groups. The control group received the usual care.

### Data analysis

The data analyses were carried out using the Statistical Package for Social Sciences (SPSS), version 20.0 (PASW ver.20, SPSS inc. Armonk, NY). The data analysis involved calculating percentage distributions and means of the gender, gestational age, birth weight, and type of feeding shown by the massage and control group of preterm infants. The homogeneity test (*X*^*2*^) and independent t-test were used for the comparison of massage and control groups. The Wilcoxon signed test was used to compare intragroup measures and was performed to evaluate the differences in terms of average frequency of defecation, daily weight gain, frequency of vomiting, GRV excess and abdominal circumference between the first day and last day of the study. The Mann–Whitney U test was used to compare intergroup measurements and compare the difference between the two groups’ the first day and last day mean values. The confidence interval of 95%; p < 0.05 was considered to be statistically significant.

### Ethical considerations

Approval to conduct the study was obtained from the Ethics Committee of Ataturk University Medical Faculty and head physician of the university hospital. After giving the necessary information to the families who had agreed to participate in the research, written permission was obtained from the families in the massage group and the control group families.

## Results

Complete data was available for 27 preterm infants. Comparison of the massage and control groups in accordance with the identifying characteristics of preterm infants included within the scope of the study is presented in Table 1. There was no statistically significant difference between groups in terms of gestational age, gender, birth weight, type of feeding, the first day measures (weight, vomiting, GRV excess, frequency of defecation or abdominal circumference).

**Table 1:** **Characteristics of preterm infants**

	**Massage group**	**Control group**	**Statistical significance**
Gestational age (week)	29.42 ± 3.13	28.23 ± 1.48	Z = −.738
			p > .05
Birth weight (g)	1218.57 ± 226.91	1172.69 ± 194.51	Z = −1.000
			p > .05
Gender			
Male n (%)	9 (64.3%)	7 (53.8%)	*X* ^*2*^ = .304
Female n (%)	5 (35.7%)	6 (46.2%)	p > .05
Type of feeding			
Mother’s milk	4 (28.6%)	2 (15.4%)	*X* ^*2*^ = .678
Mother’s milk and formula	10 (71.4%)	11 (84.6%)	p > .05

In the massage group, it was that 64.3% of the preterm infants were male and the infants’ average birth weight was 1218.57 ± 226.91 g and their average gestational age was 29.42 ± 3.13 weeks. It was found that 71.4% were being fed their mother’s milk and formula.

In the control group, it was determined that 53.8% of the preterm infants were male and the infants’ average birth weight was determined as 1172.69 ± 194.51 g and their average gestational age is determined as 28.23 ± 1.48 weeks. It was found that 84.6% were being fed their mother’s milk and formula.

In Table 2, the mean and comparison of the first day and the last day measurements for weight, vomiting, GRV excess, frequency of defecation and abdominal circumference.

**Table 2:** **Comparison of the first day and last day measurements about feeding intolerance for the massage and control group**

	**Massage group**		**p**	**Control group**		**p**
	**First day**	**Last day**		**First day**	**Last day**	
**Frequency of defecation**	1.78 ± 1.05	2.28 ± 1.13	>.05	2.07 ± 0.75	2.15 ± 0.98	>.05
**Daily weight gain**	1377.14 ± 288.85	1462.85 ± 281.96	**<.05**	1401.76 ± 316.14	1464.15 ± 325.42	**<.05**
**Frequency of vomiting**	2.14 ± 2.07	0.35 ± 0.49	**<.05**	1.46 ± 0.96	0.84 ± 0.89	>.05
**Abdominal circumference (cm)**	25.14 ± 3.77	23.21 ± 3.53	**<.05**	26.30 ± 3.56	25.84 ± 4.14	>.05
**GRV excess**	3.21 ± 4.91	0.0 ± 0.0	**<.05**	1.15 ± 2.60	0.0 ± 0.0	>.05

p < 0.05 was considered to be statistically significant.

Analysis of the Mann–Whitney U test was used to test for differences between the massage and control group’s first day measurements. After comparison of the massage group preterm infants with infants in the control group, no statistically significant difference was found between the groups in terms of weight, frequency of defecation, vomiting, abdominal circumference and GRV excess.

Analysis of the Wilcoxon signed test was used to test for differences between first day and last day average in the intra-group. When frequency of defecation measurements were analysed, the difference between the first day and last day of the study was not statistically significant in the massage group. However, when daily weight gain, frequency of vomiting, abdominal circumference and GRV excess measurements were analysed, the differences between the first day and last day of the study were statistically significant in the massage group. In this study, we found that frequency of vomiting, abdominal circumference and GRV excess of the last day measures decreased to the first day measures for massage group and the difference was found to be statistically significant (p < 0.05). Conversely, we found that frequency of defecation, and daily weight gain of the last day measures increased to the first day measures for massage group. In the control group, the differences between the first day and last day of the study were not statistically significant for parameters except for daily weight gain (Table 2).

## Discussion

Feeding intolerance is extremely common in premature infants. Therefore, it is important to know and assess correctly, the warning signs of the possible complications of enteral feeding. The most frequent signs of a suspected FI in preterm infants are the presence of gastric residuals and abdominal distension [1]. It is common practice to check the GRV before each feeding in infants [16]. According to the data obtained on the first day of the study, GRV was observed in five of the babies in the massage group (n = 14), whereas it was observed in only three of the babies in the control group (n = 13). When the data collected on the last day of the study was analysed, GRV was not observed in any of the groups. The decrease in the amount of GRV in the massage group was found to be statistically significant (Table 2).

It is reported in the literature that abdominal massage can stimulate parasympathetic activity resulting in a GI tract response [17,18]. Abdominal massage accelerates peristalsis by changing intra-abdominal pressure and creating a mechanical and reflexive effect on the intestines, decreasing abdominal distension and increasing intestinal movements [19,20]. This mechanism causes a significant shortening in the time for colonic passage [21]. The effects of abdominal massage have been assessed in a small number of clinical studies [22,23]. Lee [24] showed the effect of massage twice daily for 10 days in infants with gestational aged less than 36 weeks. The vagal tone was significantly higher after massage than before massage in the experimental group. One study of preterm neonates examined how massage influenced weight gain [8]. Vagal activity and gastric motility were measured before, during and after massage therapy sessions on the first and fifth day of massage therapy to determine whether preterm infant massage leads to consistent increases in vagal activity and gastric motility and whether these increases are associated with greater weight gain [8]. In our study, we examined this potential mechanism by assessing indices of vagal activity and gastric motility in preterm neonates receiving moderate pressure massage therapy [12]. Based on previous findings, we hypothesised that preterm neonates receiving moderate pressure massage therapy would show greater weight gain and a greater increase in vagal activity and gastric motility, although not a greater caloric intake than preterm neonates receiving light pressure stimulation or the controls. In this study, preterm neonates receiving moderate pressure massage therapy exhibited greater weight gain and increased vagal tone and increased gastric motility during and immediately after treatment. Gastric motility and vagal activity during massage therapy, in turn, were significantly related to weight gain [12,25].

We measured the abdominal circumference as one of the sign of the abdominal distension. In the massage group and the control group were compared on the first day of the study, there was no statistically significant difference between the two groups; however, a statistically significant difference was detected between the groups in the measurements done on the last day of the study.

Vomiting is the most serious complication associated with enteral feeding, increasing the risk of aspiration and pneumonia [22]. On the first day of the study, vomiting was observed in nine babies (64.3%) in the massage group, whereas this figure fell to five (35.7%) on the last day of the study. Conversely, vomiting was observed in 10 babies (76.9%) in the control group on the first day of the study and in eight babies (61.5%) on the last day of the study. The decrease in the number of vomiting instances of the babies in the massage group was determined to be statistically significant. This reduction is an expected result. The increase in the number of defecation and the decrease in the number of vomiting instances per day as a result of the decrease in abdominal distension are desired outcomes.

## Conclusions

In conclusion our study determined that abdominal massage is efficient in preventing GRV excess and abdominal distension and vomiting in enterally fed preterm infants. In accordance with the results of the study, we suggest that nurses should apply abdominal massage twice a day as an intervention helping to prevent GRV excess and abdominal distension in enterally fed preterm infants; however, more studies are needed to investigate the effect of abdominal massage on prevention of delayed gastric emptying in enterally fed infants. This study should be repeated by applying abdominal massage.
